# Supplementary material for: Predictors of Occult Metastasis and Prognostic Factors in Patients with cN0 Oral Cancer Who Underwent Elective Neck Dissection
Source: Diseases. 2024 Feb 12;12(2):39. doi: 10.3390/diseases12020039 (PMC10888440; doi:10.3390/diseases12020039)
Supplement: Supplementary file 1 [file diseases-12-00039-s001.zip › diseases-2684758-supplementary.pdf]

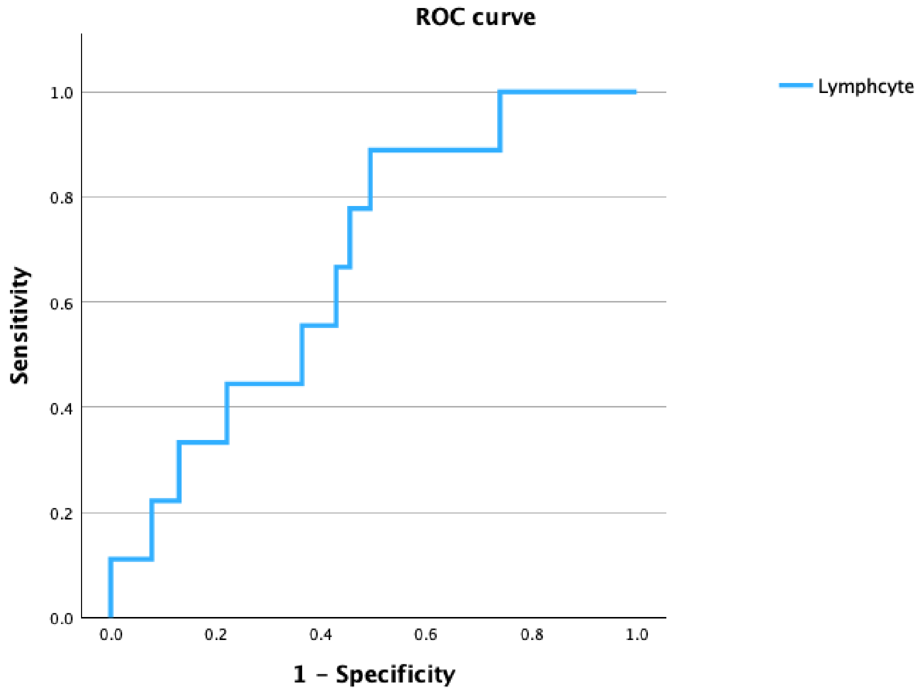

Supplementary Figure S1. Lymphocyte with occult metastasis. The area under the receiver operating characteristic (ROC) curve was 0.677 with the 95% confidence index (CI) of 0.515-0.839 (P=0.032).

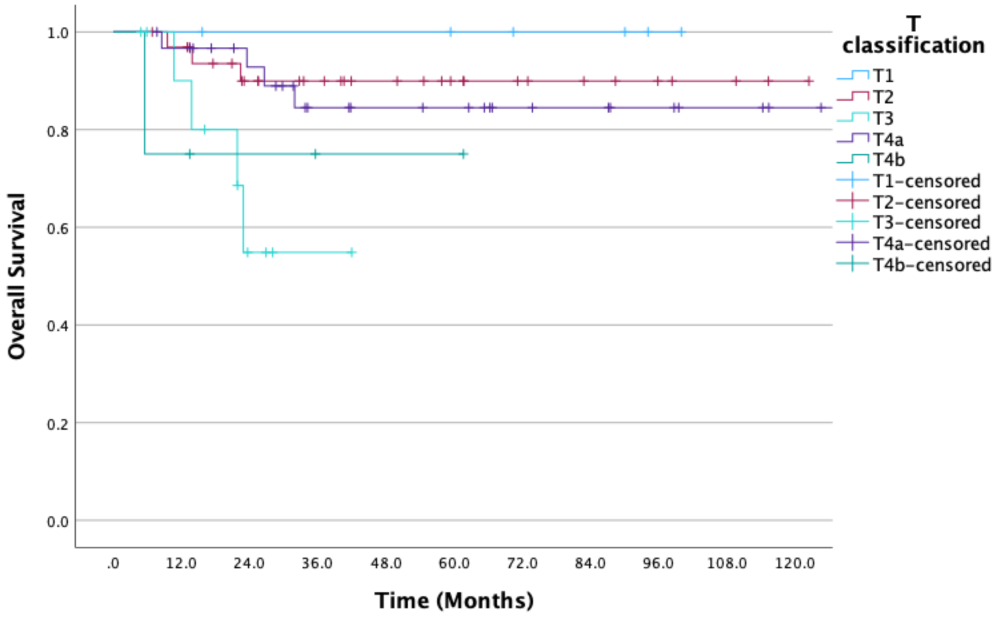

Supplementary Figure S2. Overall survival rate according to the T classification. There was a significant difference in T classification (P=0.045).

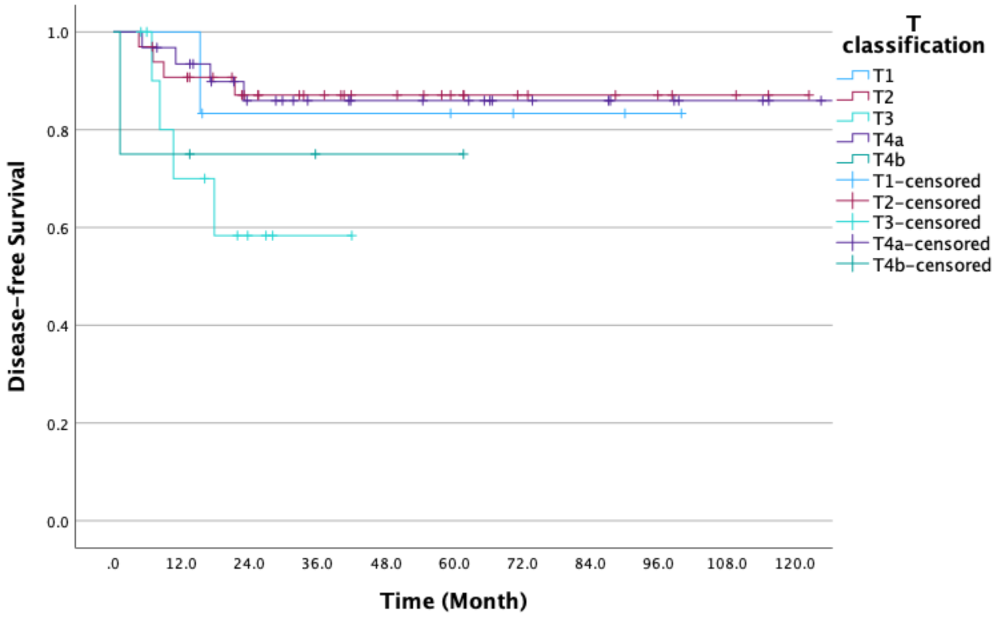

Supplementary Figure S3. Disease-free survival rate according to the T classification. There was no significant difference in the T classification.

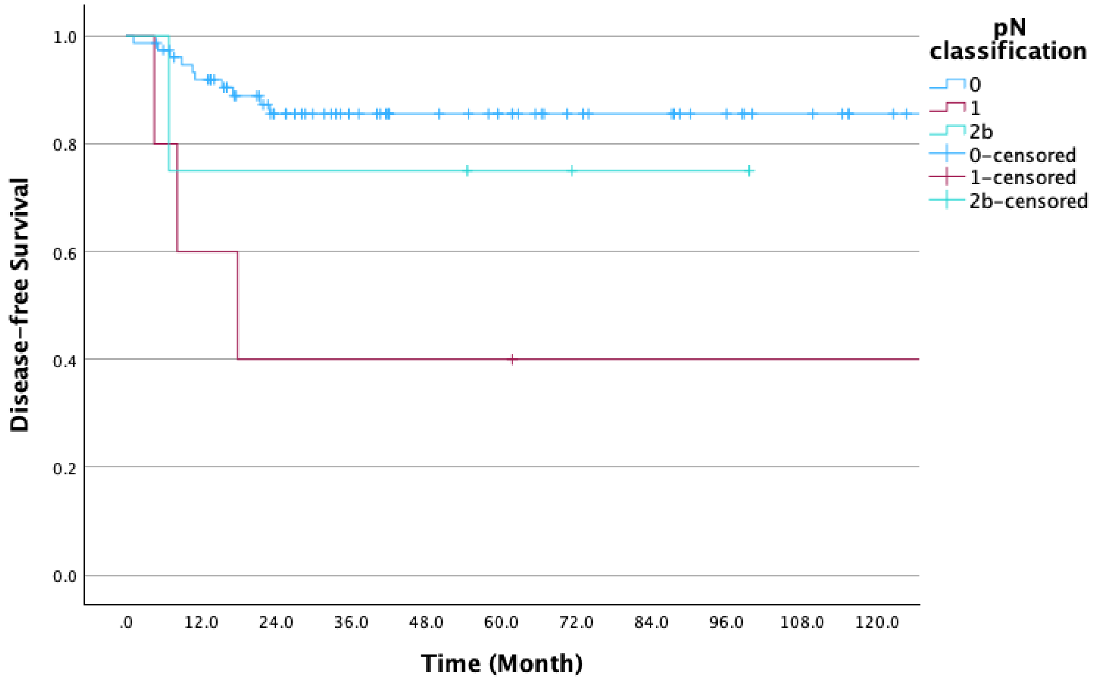

Supplementary Figure S4. Disease-free survival rate according to the pN classification. There was a significant difference in the pN classification (P=0.009).

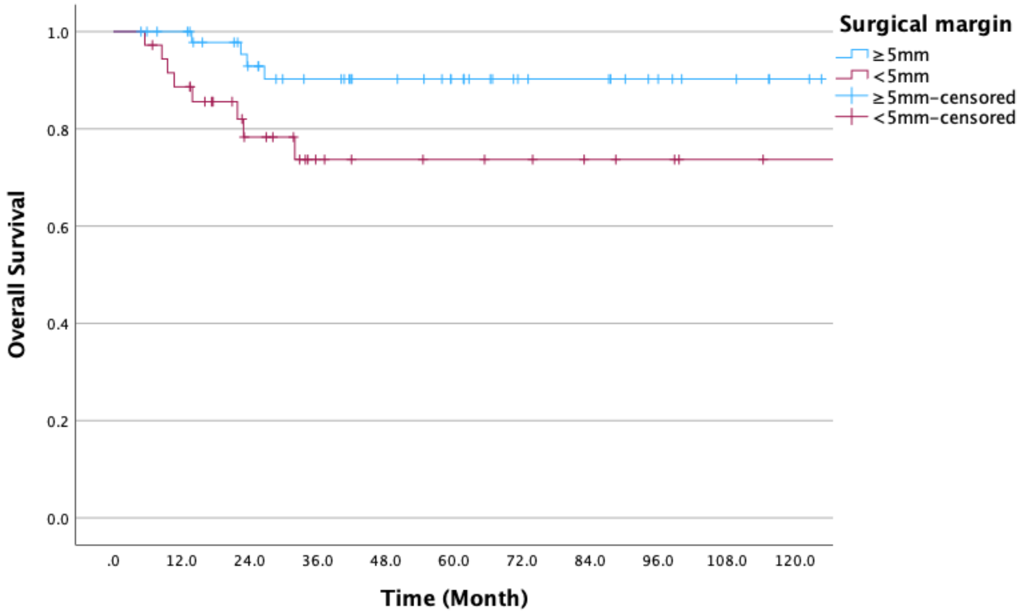

Supplementary Figure S5. Overall survival rate according to the surgical margin. There was a significant difference in surgical margin between ≥5mm and <5mm (P=0.043).

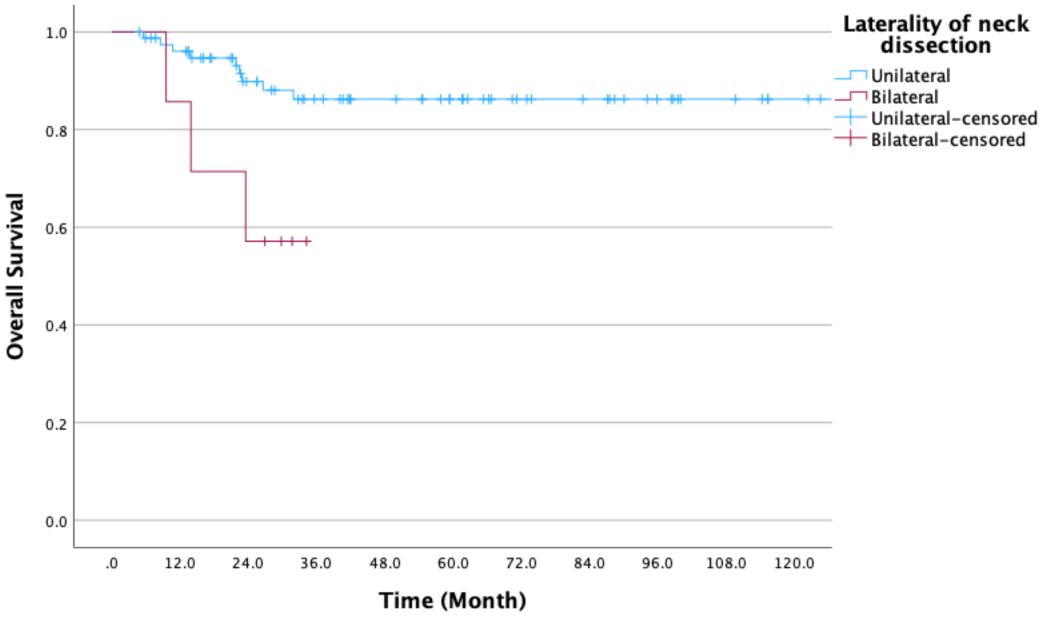

Supplementary Figure S6. Overall survival rate according to the laterality of neck dissection. There was a significant difference in the laterality of neck dissection between unilateral and bilateral (P=0.023).

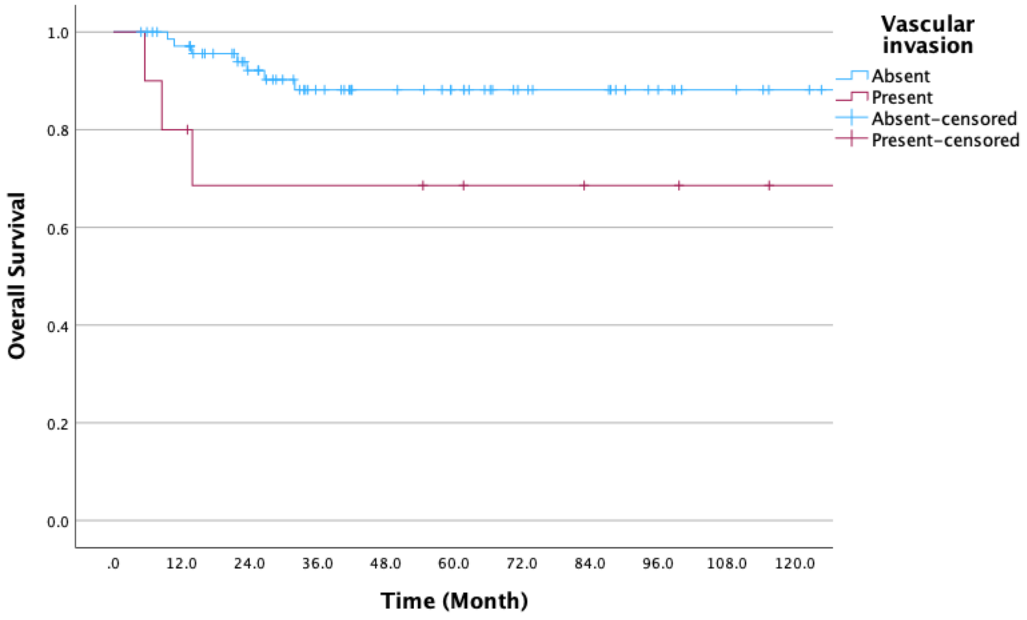

Supplementary Figure S7. Overall survival rate according to the vascular invasion. There was a significant difference in the vascular invasion between absent and present (P=0.049).

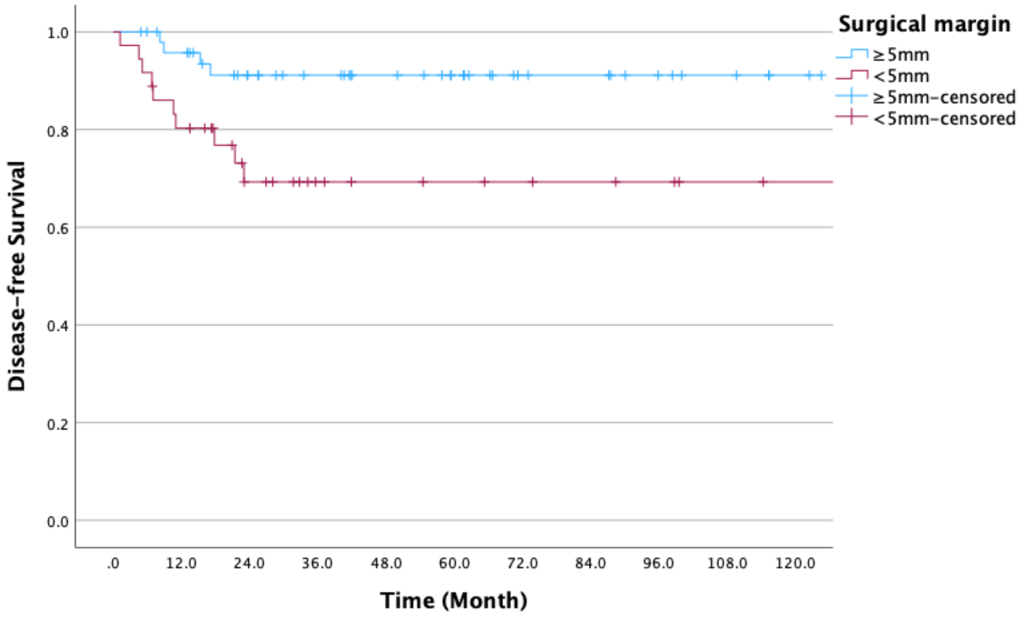

Supplementary Figure S8. Disease-free survival rate according to the surgical margin. There was a significant difference in the surgical margin between ≥5mm and <5mm (P=0.013).

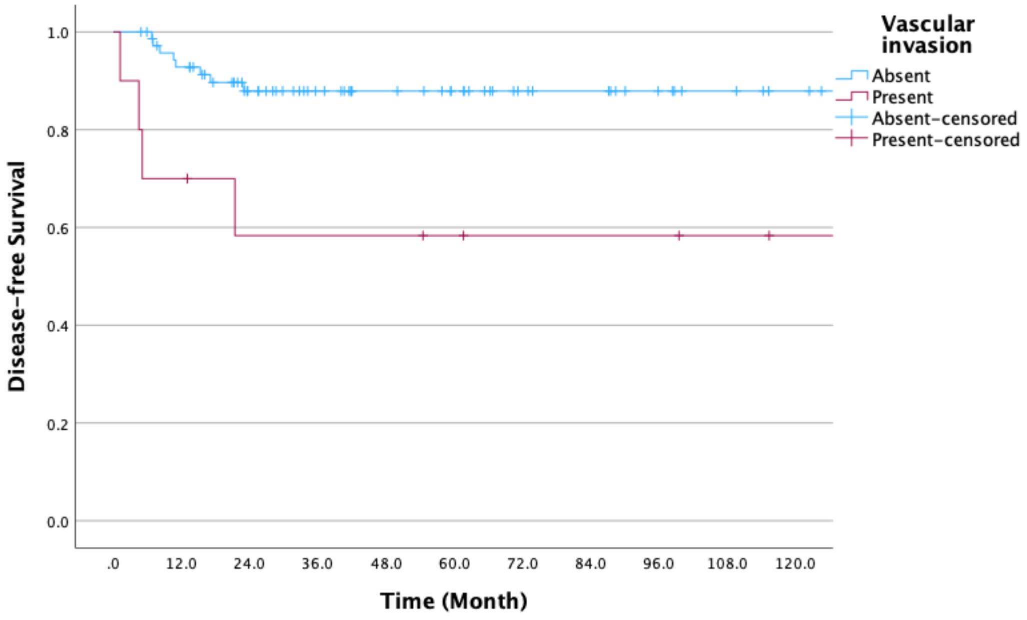

Supplementary Figure S9. Disease-free survival rate according to the vascular invasion. There was a significant difference in the vascular invasion between absent and present (P=0.006).
